# From deep brain stimulation to brain–computer interfaces: current progress in implantable neurotechnology

**DOI:** 10.3389/fnins.2026.1928223

**Published:** 2026-08-19

**Authors:** Volodymyr Mavrych, Olena Bolgova, Leen Alhamd, Rasha Alissa

**Affiliations:** 1 College of Medicine, Alfaisal University, Riyadh, Saudi Arabia; 2 College of Medicine, Sulaiman Alrajhi University, Al Bukayriyah, Alqassim, Saudi Arabia

**Keywords:** brain–computer interfaces, deep brain stimulation, neuromodulation, neuromorphic computing, speech neuroprosthesis

## Abstract

Brain implants, including deep brain stimulation (DBS) systems, brain–computer interfaces (BCIs), and speech neuroprostheses, are moving from the proof-of-concept stage to early clinical deployment. Although these systems target different clinical problems, we argue that they share a common closed-loop architecture (sensing, decoding, stimulation or output, power and telemetry, and chronic clinical validation) and that their translational pace is set by bottlenecks at these shared stages rather than by challenges unique to each technology. Recent milestones, including the regulatory clearance of a cortical interface and an expanding set of implanted-BCI trials, have accelerated progress in the field. This mini-review uses this shared architecture to compare progress across three fronts: the shift from open-to-closed-loop DBS and its widening range of neurological and psychiatric indications; BCIs restoring motor and sensory function; and speech neuroprostheses that decode attempted speech into text, voice, and facial animation. We highlight enabling advances in flexible electrodes, AI-assisted decoding, and neuromorphic edge processing, and examine unresolved controversies over electrode architecture and system design. We conclude that the same handful of bottlenecks recur across all three technologies (long-term stability, neural coding, and equitable access) and outline the governance frameworks needed alongside continued engineering progress.

## 1 Introduction

Brain implants, also termed neural implants or implanted brain–computer interfaces (iBCIs), are devices placed in or on the nervous system to record, modulate, or stimulate neural activity for therapeutic or communicative purposes (Benabid et al., 2011; Patrick-Krueger et al., 2024). These devices encompass established DBS systems along with emerging BCIs and speech neuroprostheses that support movement, communication, and closed-loop control (Patrick-Krueger et al., 2024; U.S. Food and Drug Administration, 2021). Their translational importance is growing as movement disorders, paralysis, and severe speech impairment continue to create significant unmet clinical needs (Sarica et al., 2023). Recent milestones, such as the FDA's 2025 clearance of Precision Neuroscience's cortical interface (Precision Neuroscience, 2025) and the expanding number of implanted-BCI trials (Patrick-Krueger et al., 2024), have shifted the field from proof-of-concept engineering toward early clinical deployment.

A key conceptual advance is the bidirectional interface, which not only decodes neural activity but returns information *via* targeted stimulation, enabling closed-loop communication between tissue and machine (Hughes et al., 2020). This has made AI-enabled decoding, adaptive control, and human-centered design central to development, while raising ethical questions of privacy, autonomy, safety, and equitable access. We argue that, despite their different clinical targets, DBS, BCIs, and speech neuroprostheses are converging on a common closed-loop architecture: sensing or biomarker detection, decoding, stimulation or output, power and telemetry, and chronic clinical validation (Table 1), and that the pace of clinical translation across all three is set by bottlenecks at these shared stages rather than by challenges unique to each technology. The aim of this mini-review is therefore to use this shared architecture as a framework for comparing progress across deep brain stimulation, brain–computer interfaces, and speech neuroprostheses, and for identifying the technical and ethical barriers that will determine whether these systems reach routine clinical use. Table 1 summarizes this shared architecture across the three technologies; the sections that follow are organized around it, examining in turn how each technology addresses sensing, decoding, stimulation or output, power and telemetry, and chronic clinical validation.

**Table 1:** Shared closed-loop architecture across deep brain stimulation, brain–computer interfaces, and speech neuroprostheses.

Technology	Sensing/biomarker	Decoding/control	Stimulation or output	Power and telemetry	Chronic stability/clinical validation
Deep brain stimulation	Beta-band (13–30 Hz) and other condition-specific network signatures (Neumann et al., 2016, 2023; Widge, 2024)	Threshold- or biomarker-triggered adaptive algorithms (Neumann et al., 2016, 2023)	Closed-loop electrical stimulation of subcortical/network targets (STN, GPi, Vim, ANT)	Implanted pulse generator; limited onboard sensing bandwidth	Years of use in established indications; adaptive/psychiatric applications are less mature
Brain–computer interfaces (motor/sensory)	High-density penetrating or surface arrays recording the motor/somatosensory cortex (Patrick-Krueger et al., 2024)	AI-assisted real-time decoding of movement intent (Chen et al., 2025)	Effector or spinal-interface control; ICMS for sensory feedback (Lorach et al., 2023; Greenspon et al., 2024)	Wireless bandwidth is the key bottleneck; onboard compression increasingly required	Demonstrated over months in small cohorts; one surface array was FDA-cleared for ≤ 30-day use
Speech neuroprostheses	Intracortical/ECoG recording of the speech cortex during attempted speech (Srinivasan et al., 2026)	AI decoders mapping neural activity to text/phonemes/voice (Willett et al., 2023; Card et al., 2024)	Synthesized voice, text, and facial avatar output; no stimulation feedback pathway	High-bandwidth streaming needed for low-latency naturalistic synthesis (Littlejohn et al., 2025)	Performance sustained for >8 months in one participant (Card et al., 2024); broader durability data limited

STN, subthalamic nucleus; GPi, globus pallidus internus; Vim, ventral intermediate nucleus; ANT, anterior nucleus of the thalamus; ICMS, intracortical microstimulation; ECoG, electrocorticography. This table is qualitative and illustrative, synthesized from the sources cited in the sections below, rather than being derived from a single comparative study.

## 2 Materials and methods

Although Mini Reviews do not require a formal Materials and Methods section, we summarize our literature-search strategy for transparency. We searched PubMed, Scopus, and Web of Science for English-language articles published between January 2010 and June 2026, combining controlled vocabulary and free-text terms. The reference lists of the retrieved articles were screened for additional sources. Priority was given to peer-reviewed clinical studies, randomized trials, and recent reviews; preprints, conference abstracts, and unpublished data were excluded, and regulatory decisions were confirmed against primary agency or manufacturer announcements.

**Search string used:** *(“deep brain stimulation” OR “brain–computer interface” OR “brain-machine interface” OR “neural implant” OR “speech neuroprosthesis” OR “neuroprosthetic”) AND (“closed-loop” OR “adaptive” OR “decoding” OR “electrode” OR “neuromorphic” OR “clinical trial” OR “restoration”)*

## 3 Deep brain stimulation: evolution and emerging applications

Within the framework introduced in Table 1, DBS is the most clinically mature instance of closed-loop implantable neurotechnology, and it best illustrates most directly how advances in biomarker sensing and adaptive stimulation translate into approved indications.

### 3.1 From open-loop to closed-loop systems

**Figure 1:**
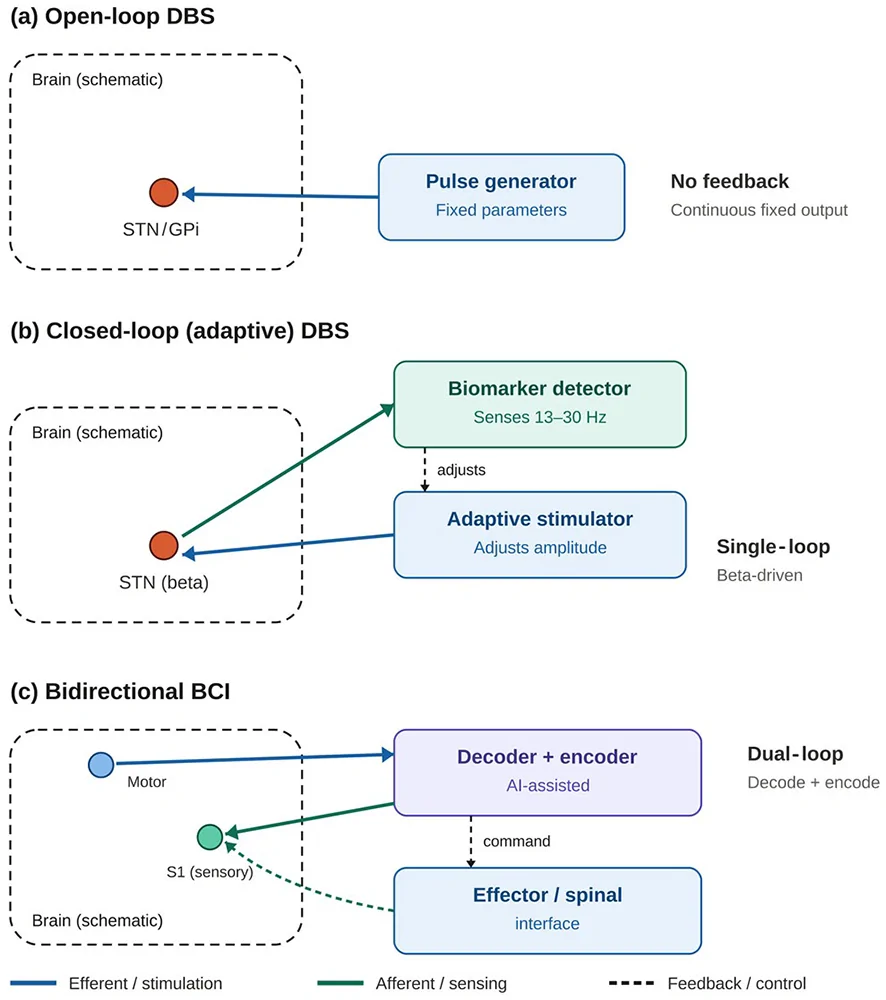
Schematic comparison of open-loop, closed-loop, and bidirectional neuromodulation. **(a)** Open-loop deep brain stimulation (DBS): a pulse generator delivers continuous, fixed-parameter stimulation to a subcortical target (e.g., STN, GPi), with no sensing and no feedback loop. **(b)** Closed-loop (adaptive) DBS: a biomarker detector senses beta-band oscillations (13–30 Hz) from the target and adjusts an adaptive stimulator in real time, forming a single feedback loop. **(c)** Bidirectional brain–computer interface (BCI): an AI-assisted decoder–encoder reads motor cortex activity to drive an effector or spinal interface, while the encoder stimulates the somatosensory cortex (S1) to restore sensory feedback, forming a dual feedback loop (decode + encode). Solid blue arrows indicate efferent/stimulation pathways; solid teal arrows indicate afferent/sensing pathways; dashed arrows indicate internal feedback or control signals. The brain outlines are simplified schematics, not anatomically precise renderings. This schematic is conceptual and illustrative rather than derived from measured data; the panels represent generalized device architectures and feedback loops.

Conventional DBS relies on open-loop paradigms, delivering continuous stimulation to predefined subcortical targets regardless of clinical state (Ghasemi et al., 2018) (Figure 1a). In movement disorders, particularly Parkinson's disease (PD), the subthalamic nucleus (STN), and globus pallidus internus (GPi) remain the principal targets, producing sustained motor improvement (Macerollo et al., 2020). However, continuous stimulation ignores temporal variability in symptoms and neural activity, potentially causing suboptimal efficacy, side effects, and unnecessary energy use (Wang et al., 2023). Adaptive DBS (aDBS) addresses this by incorporating real-time input to dynamically modulate stimulation (Neumann et al., 2016, 2023; de Neeling et al., 2026) (Figure 1b). aDBS relies on neural biomarkers, most notably pathological beta-band oscillations (13–30 Hz) from the STN, which correlate with motor impairment in PD (Neumann et al., 2016, 2023; Tinkhauser et al., 2017), to adjust stimulation intensity as the disease state fluctuates, improving precision and reducing adverse effects (Little et al., 2014).

Beyond movement disorders, condition-specific neural signatures are being identified in neuropsychiatric diseases, extending closed-loop DBS to depression and obsessive–compulsive disorder (OCD) (Widge, 2024; Chiu et al., 2026). These advances reflect a shift toward network-informed neuromodulation guided by individualized circuit dysfunction, marking a critical step toward more efficient, personalized, and physiologically responsive therapy.

### 3.2 Expanding clinical applications

DBS has been shown to be effective in treating several neurological and psychiatric conditions, and it has received regulatory approval in well-defined indications (Coenen et al., 2015). Representative clinical evidence across these indications, alongside landmark BCI and speech-neuroprosthesis studies, is summarized in Table 2. In PD, STN or GPi stimulation significantly improves motor symptoms and selected non-motor features (Odekerken et al., 2016). In essential tremor, ventral intermediate nucleus (Vim) stimulation substantially reduces tremor (Sun et al., 2024). In dystonia, GPi stimulation progressively improves tone and abnormal postures over weeks to months (Khanom et al., 2023). DBS is also approved for treatment-resistant OCD and refractory epilepsy, with stimulation of cortico-striatal-thalamic circuits and the anterior nucleus of the thalamus, respectively, reducing symptoms and seizure frequency (Mar-Barrutia et al., 2021; Herrman et al., 2018).

**Table 2:** Representative clinical and landmark evidence from the domains of deep brain stimulation, brain–computer interface, and speech-neuroprostheses.

Domain	Indication/ function	Target/interface	Representative study	Design	Key outcome
DBS	Parkinson's disease (conventional and adaptive)	STN/Gpi	Odekerken et al., 2016; de Neeling et al., 2026	RCT; review	Significant motor improvement; aDBS improves precision and reduces adverse effects vs. continuous stimulation
DBS	Essential tremor	Vim thalamus	Sun et al., 2024	RCT	Significant tremor reduction; informs target selection
DBS	Cervical dystonia	Gpi	Khanom et al., 2023	Cohort (5-year)	~57% improvement in TWSTRS motor scores
DBS	Refractory epilepsy	Anterior nucleus of the thalamus	Herrman et al., 2018	RCT (double-blind)	Reduced seizure frequency; ≥50% reduction in a subset
DBS	Treatment-resistant OCD	Cortico-striatal-thalamic circuits	Mar-Barrutia et al., 2021	Systematic review (20 years)	Symptom reduction; established indication
DBS	Investigational: depression; Alzheimer's disease	Mood-regulating networks; fornix/nucleus basalis of Meynert	Wu et al., 2021; Picton et al., 2023	Meta-analysis; systematic review	Preliminary antidepressant/memory effects; placebo-controlled confirmation needed
BCI/speech	Chronic tetraplegia (SCI)	Cortical + spinal (“digital bridge”)	Lorach et al., 2023	Case study	Standing, walking, stair climbing; naturalistic gait
BCI/speech	Cervical SCI (hand grasp)	Subdural surface electrodes + FES	Cajigas et al., 2021	Case study	89.0% mean movement-intent decoding accuracy
BCI/speech	Somatosensory restoration	Intracortical microstimulation	Greenspon et al., 2024	Case study	Stable, precise evoked tactile percepts
BCI/speech	ALS (speech-to-text)	Intracortical speech cortex	Willett et al., 2023; Card et al., 2024	Case study	62–97.5% accuracy, depending on the system; sustained for >8 months in one participant
BCI/speech	Severe limb/vocal paralysis (text, voice, avatar)	High-density ECoG	Metzger et al., 2023; Littlejohn et al., 2025	Case study	78 wpm, 25% WER; low-latency, naturalistic synthesis
BCI/speech	Bilingual speech decoding	High-density ECoG	Silva et al., 2024b	Case study	Shared articulatory representations across languages

STN, subthalamic nucleus; GPi, globus pallidus internus; Vim, ventral intermediate nucleus; OCD, obsessive–compulsive disorder; TWSTRS, Toronto Western Spasmodic Torticollis Rating Scale; SCI, spinal cord injury; FES, functional electrical stimulation; ALS, amyotrophic lateral sclerosis; ECoG, electrocorticography; wpm, words per minute; WER, word error rate; RCT, randomized controlled trial. The evidence level varies across rows (RCTs, cohort studies, systematic reviews, meta-analyses, and single-participant case studies); entries should be interpreted within their respective methodological context.

Despite these indications, access remains uneven, with geographic and socioeconomic disparities shaping who receives neuromodulation (Moens et al., 2024). The scope is also expanding into investigational indications. For example, in treatment-resistant depression, early open-label studies targeting mood networks have reported antidepressant effects; however, placebo-controlled trials remain necessary (Wu et al., 2021). In Alzheimer's disease, DBS of the fornix or nucleus basalis of Meynert is under investigation as a way to modulate memory circuits, although evidence remains preliminary, with small sample sizes, heterogeneous outcomes, and uncertain durability (Picton et al., 2023).

### 3.3 Novel stimulation paradigms

Recent work aims to improve efficacy while reducing side effects and energy demand. Because continuous high-frequency stimulation may not match the dynamic properties of neural circuits, alternative paradigms, such as burst-cycling stimulation, have been designed to engage physiological oscillations and modulate pathological network activity (Wong et al., 2021). In parallel, multimodal strategies that combine invasive and non-invasive techniques are emerging: non-invasive modalities, such as transcranial magnetic stimulation and focused ultrasound, are being explored for network mapping and adjunctive therapy, potentially improving target selection alongside implanted systems (Micera and Foffani, 2024). Together, these innovations underscore a shift toward more adaptive, efficient, and integrative neuromodulation.

## 4 BCIs for motor and sensory restoration

A bidirectional BCI extends the closed-loop principle introduced above by coupling a decoded motor output with an encoded sensory return, forming a dual feedback loop, as opposed to the single loop used in adaptive DBS (Figure 1c). Within the shared architecture in Table 1, motor and sensory BCIs primarily test the decoding and stimulation/output stages, under far tighter bandwidth constraints than DBS. Progress in BCIs has been driven by advances in electrode architecture, particularly efforts to increase channel count while improving biocompatibility and reducing surgical burden. High-density penetrating systems, such as Neuralink's N1, use thousands of recording electrodes (Patrick-Krueger et al., 2024), whereas non-penetrating strategies, such as Precision Neuroscience's Layer 7, use a thin, flexible cortical-surface array, which was cleared by the FDA in 2025 for temporary implantation of up to 30 days (Precision Neuroscience, 2025). Although these platforms differ in invasiveness and geometry, both highlight a central challenge: raw neural data can be generated much faster than the available bandwidth for safe, real-time, wireless transmission, making onboard compression, feature extraction, and AI-assisted decoding essential for clinical deployment (Chen et al., 2025). Landmark demonstrations across motor, sensory, and speech domains are summarized in Table 2 above. Although these landmark studies demonstrate technical feasibility, much of the current evidence derives from single-participant or very small cohorts; therefore, reported decoding metrics should be interpreted as proof of concept rather than evidence of reproducible population-level performance.

Motor restoration provides the clearest evidence that BCIs can translate neural intent into function. A clinically transformative advance is the brain-to-spine “digital bridge,” in which cortical signals from the motor cortex are decoded in real time to stimulate spinal circuits, bypassing the injury site; this technology enabled standing, walking, stair climbing, and improved independence in a man with chronic tetraplegia (Lorach et al., 2023). Subdural surface-electrode approaches further broaden clinical reach by enabling volitional motor control (such as hand grasp) from cortical recordings in patients with severe paralysis (Cajigas et al., 2021).

A key limitation of early BCIs was their one-way design, which decoded intent without restoring sensory feedback. Intracortical microstimulation is closing this gap: human studies show that somatosensory-cortex stimulation can evoke stable, precise tactile sensations, supporting more accurate object handling (Greenspon et al., 2024). In parallel, AI-driven implants are being developed for visual and auditory restoration (van Stuijvenberg et al., 2026), while cochlear implants remain the most successful sensory neuroprosthesis and the clearest benchmark for future bidirectional systems (Wilson, 2017).

## 5 Speech neuroprostheses

In the framework outlined in Table 1, speech neuroprostheses represent the most advanced decoding-to-output pipeline among the three technologies. However, current systems remain one-directional, providing no stimulation-based feedback to the user, unlike DBS and sensory BCIs.

Speech neuroprostheses address one of the most disabling consequences of neurological injury (loss of speech) and aim to restore communication that is faster and more expressive than text-only assistive systems (Silva et al., 2024a). Progress has been driven by improved recording coverage, decoding strategies, and long-term stability, enabling speech-related cortical activity to be translated into text, synthesized voice, and facial movement (Silva et al., 2024a).

A significant advance is rapid calibration with high, durable accuracy. In one participant with ALS, an intracortical neuroprosthesis achieved 97.5% word accuracy across a 125,000-word vocabulary over a period of more than 8 months (Card et al., 2024) (Table 2). In another participant with severe limb and vocal paralysis, high-density recordings enabled real-time multimodal decoding of text, speech audio, and facial avatar animation, reaching a median of 78 words per minute with a 25% word error rate, with synthesized speech personalized to their pre-injury voice (Metzger et al., 2023). An earlier high-performance system decoded attempted speech at 62 words per minute, with a 9.1% word error rate using a 50-word vocabulary, and at 23.8% using a 125,000-word vocabulary (Willett et al., 2023). Maintaining performance over months strengthens the clinical relevance of these systems for individuals living with paralysis or anarthria.

Our understanding of the neural basis has also improved: speech-related signals concentrate in the ventral precentral gyrus and the adjacent sensorimotor cortex, and these signals are often detectable during attempted speech, even without vocal output (Srinivasan et al., 2026). This “attempted speech” substrate reflects the intention to speak rather than overt articulation. In bilingual users, overlapping cortical representations may support neuroprostheses that operate across languages (Silva et al., 2024b). Current work is shifting toward real-time synthesis and naturalistic communication, with streaming brain-to-voice output generated at low latency and facial avatar control that adds visual and non-verbal expression (Littlejohn et al., 2025). Together, these developments show speech neuroprostheses moving from experimental decoders toward clinically meaningful interfaces that can restore both language output and personal expression.

## 6 Neuromorphic computing and signal processing

This section addresses the power-and-telemetry stage of the shared architecture in Table 1, a constraint common to DBS, BCIs, and speech neuroprostheses alike.

Neuromorphic computing is emerging as an energy-efficient framework for edge signal processing, where local computation reduces bandwidth, latency, and power (Choi, 2024). By moving inference closer to the sensor, neuromorphic systems avoid transmitting large volumes of raw data, which is important for real-time biomedical applications (Tian et al., 2023). Event-driven processing, which triggers computation only when new input events occur, suits the asynchronous, sparse signals typical of neural recordings and reduces the telemetry burden in compact, closed-loop devices (Yao et al., 2024).

Alongside neuromorphic hardware, machine learning methods remain essential for decoding and adaptation. Spiking neural networks offer an alternative, brain-inspired architecture that is well-suited to the temporal, event-based structure of neural signals (Choi, 2024). Because signal characteristics drift over time due to electrode displacement, encapsulation, device aging, and genuine within-subject physiological variation (Dunlap et al., 2020), continual learning enables incremental adaptation without full retraining, while federated learning supports model training across devices without centralizing private data, an advantage for clinical and wearable use (Hu et al., 2021). Together, these approaches mark a shift from static pipelines toward adaptive, decentralized, energy-aware systems for next-generation neural decoding.

## 7 Discussion

The synthesis below is organized around the chronic stability and clinical validation stages of the framework in Table 1, the two axes on which all three technologies remain least mature.

**Figure 2:**
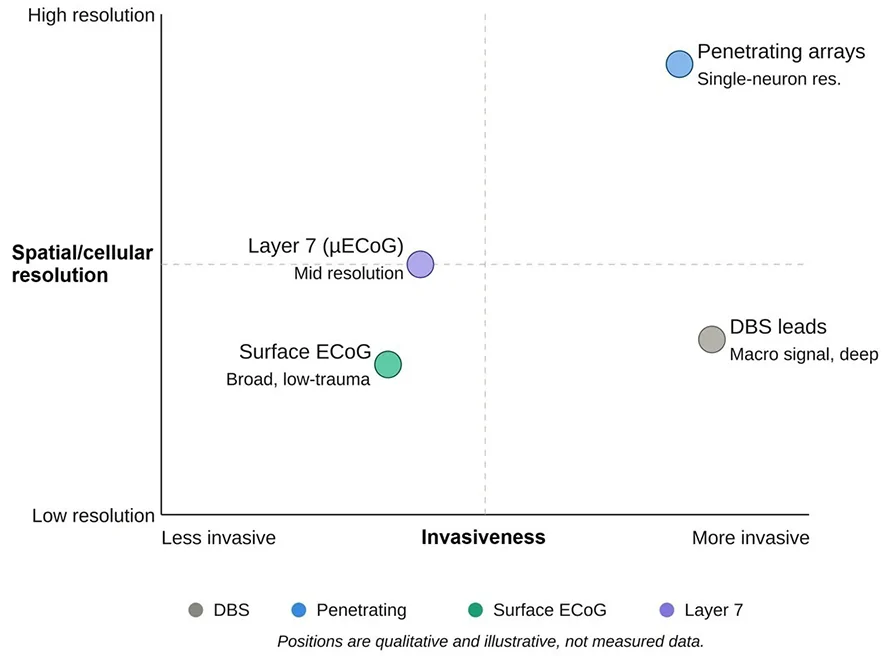
Electrode architectures plotted by invasiveness (*x*-axis) vs. spatial/cellular resolution (*y*-axis). DBS depth leads and penetrating microelectrode arrays (e.g., Utah arrays, Neuralink N1 threads) are at the higher end of the invasiveness scale, with penetrating arrays reaching single-neuron resolution, while DBS leads capture macroscale signals from deep targets. Surface ECoG and Precision Neuroscience's Layer 7 micro-ECoG are less invasive, with Layer 7 achieving somewhat finer resolution than conventional surface ECoG. No electrode architecture currently combines low invasiveness with high resolution, illustrating the central design trade-off discussed in the text. The dot positions are qualitative and illustrative, based on the relative characteristics described in the literature rather than on measured data from a single study.

The development of brain implants is shaped by unresolved technical and ethical tensions. A central debate concerns penetrating vs. non-penetrating electrodes (Figure 2). High-density penetrating systems can achieve single-neuron resolution; however, they raise long-term biocompatibility concerns, as rigid probes may cause inflammation and glial scarring, which degrade signal quality (Li and Yang, 2025). Surface-based systems, such as Layer 7, reduce tissue trauma, but they offer less depth access and lower cellular resolution (Lehner et al., 2026). Progress in flexible, stretchable bioelectronics may reduce this mechanical mismatch; however, such devices introduce their own insertion challenges (Li and Yang, 2025). No single architecture yet balances signal fidelity, safety, and durability, so the optimal design remains application-specific.

A second controversy concerns the difference between fully implantable vs. percutaneous systems. Fully wireless, battery-free devices better suit naturalistic daily use and may reduce infection risk, but wireless transmission remains a bottleneck for high-bandwidth neural data (Won et al., 2021). Percutaneous systems provide higher-fidelity acquisition but restrict mobility and create a persistent infection route. Fully implantable high-bandwidth systems will likely be necessary for long-term home use and broader adoption.

Regulatory pathways add a further layer of complexity that is often underappreciated in engineering literature. Devices such as Precision Neuroscience's Layer 7 have advanced through the clearance process for short-term, time-limited implantation, a route that is substantially less demanding than the evidence required for permanent, chronic implants (Picton et al., 2023). As BCIs move from days-long temporary use toward years-long chronic deployment, sponsors will need long-duration safety and efficacy data that the majority of current studies, by design, do not yet provide. Aligning academic proof-of-concept timelines with the multi-year evidence base that regulators will reasonably require is therefore a translational bottleneck in itself, distinct from any single technical limitation described above.

A cross-cutting theme is the field's growing dependence on artificial intelligence. Whether the application is adaptive DBS, motor decoding, or speech synthesis, performance increasingly rests on machine-learning models that must operate on noisy, nonstationary signals in real time. This dependence is double-edged. AI-assisted decoding is what allows high-density arrays to remain useful despite bandwidth limits, and what makes rapid calibration and naturalistic output possible; however, decoders remain vulnerable to signal drift, electrode displacement, and physiological variation that accumulate over months and years (Dunlap et al., 2020). Continual and federated learning offer partial solutions by allowing models to adapt incrementally and to train across users without pooling raw neural data (Hu et al., 2021). However, neither approach fully resolves the underlying problem: a decoder that performs well in a supervised laboratory session may degrade unpredictably during unsupervised home use. Characterizing how these models fail, and how failures are detected and corrected, is as important to safe deployment as the electrode hardware itself.

Questions of corporate responsibility are also becoming increasingly important as commercial neurotechnology nears routine use. When implanted devices require maintenance, software support, or replacement, responsibility cannot end when a trial concludes, particularly because removal may carry neurological risks, and device function may become integrated into communication, autonomy, or identity (Higgins et al., 2026). The rise of neurorights adds further complexity, as legal frameworks increasingly address mental privacy and cognitive liberty while AI-enhanced decoding improves the inference of internal states (Szoszkiewicz and Yuste, 2025). Continuous neuromodulation raises related concerns about agency and selfhood, because adaptive systems that influence movement, mood, or behavior may blur the distinction between patient intention and device-mediated effect. These concerns do not argue against brain implants, but they do underscore the need for governance that evolves with the technology.

Equity is a further concern that cuts across every modality. DBS, the most mature of these therapies, already shows marked geographic and socioeconomic disparities in who receives it (Sarica et al., 2023; Moens et al., 2024), and there is little reason to expect newer, more expensive implanted BCIs to be distributed more evenly without deliberate effort. Equity concerns begin at the stage of evidence generation: if early validation cohorts remain narrowly sampled, the field may face a dual equity problem of unrepresentative development cohorts followed by unequal clinical access. Future studies should therefore report demographic composition and evaluate whether participant samples adequately reflect the populations intended to benefit from these technologies. High device costs, specialized surgical and programming expertise, and the need for long-term technical support concentrate access in well-resourced academic centers. If commercial neurotechnology follows the trajectory of other high-cost medical devices, the patients with the greatest unmet need—those with advanced paralysis, anarthria, or refractory disease in under-resourced settings—may be the last to benefit. Addressing equity therefore requires not only cheaper and more robust hardware but also reimbursement pathways, data-governance frameworks, and explicit post-trial commitments that are planned before, rather than after, wider deployment (Higgins et al., 2026).

### 7.1 Research gaps

Several gaps limit translation. First is long-term device stability: many systems perform well over weeks or months, but clinically useful implants must remain reliable for years, and signal deterioration from encapsulation, micromotion, material fatigue, or displacement is compounded in flexible probes by implantation difficulty (Li and Yang, 2025). Standardized benchmarks for chronic performance and failure reporting are still lacking, and negative or null results, such as an implant that fails to reach the expected level of performance or degrades faster than anticipated, are rarely published, leaving an incomplete picture of real-world reliability across the field. A second gap is the incomplete understanding of neural coding: robust correlations between neural signals and intended movement, speech, or sensation exist, but the underlying encoding principles are only partially understood, limiting decoder generalization and, for stimulation, rational closed-loop design. Patient heterogeneity adds further difficulty; personalization improves performance but complicates standardization and comparability. This issue is amplified by inconsistent outcome measures across BCI research. Patient heterogeneity should be considered not only a challenge to standardization, but also a biologically meaningful source of variance that may inform decoder and stimulation design. Future studies should examine whether biomarkers such as beta-band activity and responses to stimulation vary systematically across sex, age, and other demographic subgroups.

### 7.2 Future directions

The most immediate priority is the development of fully implantable, high-bandwidth, wireless systems, enabled by low-power electronics, inductive power transfer, and neuromorphic local processing that compresses features before transmission. Optogenetics offers cell-type-specific modulation with high temporal precision, but requires solutions for safe gene delivery, durable expression, immunological safety, and deep-target light delivery before human use. Predictive closed-loop systems may represent the next significant advance, using real-time biomarkers and computational models to anticipate tremor, seizures, or mood instability before full clinical expression, shifting neuromodulation from reactive to anticipatory control. Such predictive control is likely to be especially consequential in psychiatry, where the relevant biomarkers of mood, craving, and compulsion are less well-defined than the motor signatures used in Parkinson's disease, and where the boundary between therapeutic modulation and the unwanted alteration of affect or agency is correspondingly harder to draw (Widge, 2024; Chiu et al., 2026); progress in this area will depend as much on identifying reliable, individualized neural signatures as on stimulation hardware. Convergence with regenerative medicine, through biohybrid and tissue-integrated interfaces, may reduce chronic foreign-body responses and improve long-term integration. More speculative directions, including cognitive enhancement and bidirectional brain-computer-brain interfaces, remain exploratory but already highlight the need for ethical frameworks that extend beyond therapy. More broadly, the convergence of adaptive algorithms, flexible bioelectronics, and low-power neuromorphic processing points toward implants that are not only smaller and longer-lasting but also capable of learning and adjusting to each user over time - an architecture that increasingly blurs the historical distinction between passive recording devices and active therapeutic agents.

## 8 Conclusion

Brain implants are approaching a decisive stage. Their clinical benefit is established in selected settings, such as DBS for movement disorders, BCIs for motor restoration, and speech neuroprostheses for severe communication impairment, and recent milestones indicate movement beyond the proof-of-concept stage toward early clinical implementation. However, significant barriers remain: long-term stability, biocompatibility, and signal fidelity are not established at the timescales required for routine chronic use; neural coding and stimulation mechanisms are incompletely understood; and ethical concerns, including neurorights, post-trial responsibility, cost, and unequal access, remain insufficiently addressed. As Table 1 illustrates, these barriers cluster at the same architectural stages across DBS, BCIs, and speech neuroprostheses, reinforcing the idea that these are translational bottlenecks shared across the field rather than problems unique to any single technology. The next phase will likely be driven by the convergence of AI-assisted decoding, flexible bioelectronics, neuromorphic processing, and adaptive closed-loop control. However, technical progress alone will not determine the future of this field: sustained impact will require rigorous validation, standardized outcomes, durable governance, and an explicit commitment to equitable access. If these advance alongside engineering innovation, brain implants may become a transformative platform for restoring movement, communication, and autonomy. However, if not, the field risks advancing in capability faster than in accountability.
